# Endogenous CD155 drives metabolic reprogramming via PI3K/AKT/HIF‐1α‐glycolysis axis to mediate anti‐PD‐1 resistance in non‐small cell lung cancer

**DOI:** 10.1002/ctm2.70741

**Published:** 2026-08-10

**Authors:** Wei‐Guang Du, Xi‐Yang Tang, Yu‐Long Zhou, Run‐Ze Zhang, Zhi‐Bo Feng, Meng‐Chao Li, Jun‐Yang Pan, Yao Lv, Xiao‐Liang Xu, Xiao‐Long Yan, Nan Ma, Jin‐Bo Zhao

**Affiliations:** ^1^ Department of Thoracic Surgery Tangdu Hospital Fourth Military Medical University Xi'an Shaanxi China; ^2^ Department of Ophthalmology Tangdu Hospital Fourth Military Medical University Xi'an Shaanxi China

**Keywords:** AAV9_shCD155, anti‐PD‐1 therapy, CD155, glycolysis, mechanism of resistance, NSCLC

## Abstract

**Objective:**

Anti‐PD‐1 therapy resistance remains a critical barrier in non‐small cell lung cancer (NSCLC) management, and the underlying mechanisms are incompletely defined.

**Methods:**

We generated CD155‑knockout (KO) NSCLC cell lines using the CRISPR‑Cas9 system and performed systematic multi‑omics analyses, including single‑cell RNA‑seq, bulk RNA‑seq, proteomics, and metabolomics. The key molecular mechanisms were further validated by immunohistochemistry (IHC), western blotting, and chromatin immunoprecipitation (ChIP). Functional assays assessed cell proliferation, migration, and metabolic phenotypes, while the therapeutic efficacy was assessed in vivo using AAV9_shCD155.

**Results:**

Single‐cell sequencing revealed aberrantly high CD155 expression in NSCLC patients with poor response to anti‐PD‐1 therapy. High CD155 expression in NSCLC tissues correlated with unfavourable prognosis. ETS1 was identified as a direct transcriptional driver of CD155. Multi‐omics analysis and functional assays demonstrated that CD155 upregulates the expression of key glycolytic proteins (GLUT1, GLUT3, LDHB) by activating the PI3K/AKT/HIF‐1α signalling axis, thereby driving glycolytic metabolism, proliferation, and migration of tumour cells. CD155 knockout significantly suppressed these malignant phenotypes. In xenograft mouse models, monotherapy with AAV9_shCD155 effectively inhibited tumour growth and postoperative recurrence. More importantly, in humanised mouse models, combining AAV9_shCD155 with pembrolizumab produced synergistic anti‐tumour effects, more significantly suppressing tumour growth and promoting immune cell infiltration into the tumour microenvironment.

**Conclusion:**

CD155 mediates anti‐PD‐1 resistance by activating PI3K/AKT/HIF‐1α‐driven glycolytic reprogramming. Targeting CD155 combined with anti‐PD‐1 overcomes resistance, supporting a dual‐target therapeutic strategy.

**Key points:**

- CD155 is identified as a key driver of anti‐PD‐1 resistance in NSCLC.
- CD155 promotes tumour glycolysis and malignant progression via the PI3K/AKT/HIF‐1α signalling axis.
- AAV9_shCD155 combined with anti‐PD‐1 markedly inhibits tumour growth and promotes immune infiltration.

## Graphical abstract

1. CD155 is identified as a key driver of anti‐PD‐1 resistance in NSCLC.
2. CD155 promotes tumour glycolysis and malignant progression via the PI3K/AKT/HIF‐1α signalling axis.
3. AAV9_shCD155 combined with anti‐PD‐1 markedly inhibits tumour growth and promotes immune infiltration.

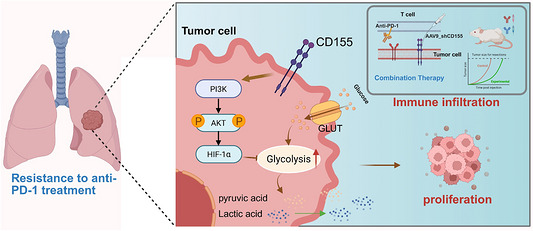

## 1 INTRODUCTION

Lung cancer remains a predominant causes of cancer‐related mortality globally.^1^ The emergence of immune checkpoint inhibitors (ICIs) has greatly advanced cancer treatment. For example, anti‐PD‐1 drugs including pembrolizumab and nivolumab have achieved significant clinical efficacy in lung cancer.^2^ Unfortunately, more than half of patients develop resistance to anti‐PD‐1 therapy.^3^

The resistance issue may be caused by other immune checkpoints. Immune checkpoint proteins mediate tumour immune evasion through various pathways, and ICIs restore tumour immune regulation by inhibiting co‐inhibitory immune checkpoint proteins.^4^ Currently, the use of anti‐PD‐1/PD‐L1 inhibitors alone seems to be insufficient to modulate the tumour immune microenvironment co‐mediated by the known 17 checkpoint pairs. Consequently, some patients with low PD‐1/PD‐L1 levels fail to respond to PD‐1/PD‐L1‐directed therapy.^5^, ^6^ A compensatory relationship seems to exist among the expression of other immune checkpoints. This drives the upregulation of alternative checkpoint receptors – including TIM‐3, TIGIT, VISTA, and LAG‐3 – as resistance to PD‐1/PD‐L1 blockade develops.^7^ For example, TIM‐3 elevation is mainly seen in T‐cell subsets that bind therapeutic PD‐1 antibodies. When PD‐1 expression is absent, this directly results in increased TIM‐3 expression, which further exacerbates resistance to anti‐PD‐1/PD‐L1 therapy.^8^ Moreover, elevated expression of other immune checkpoints on tumour cells (including FGL‐1, CD155, and SIRPA) can not only effectively inhibit immune activation by anti‐PD‐1/PD‐L1 inhibitors but also promote tumour malignancy, and thereby drive resistance to PD‐1/PD‐L1 therapy.^9^, ^10^, ^11^ Of note, similar resistance phenotypes have also been attributed to non‐coding RNA networks, including the miR‐23a/27a/24‐2 cluster in NSCLC.^12^ In this study, we found that CD155 serves a critical function in resistance to anti‐PD‐1 therapy. Nevertheless, its precise mode of action in NSCLC remains to be fully defined.

**FIGURE 1:**
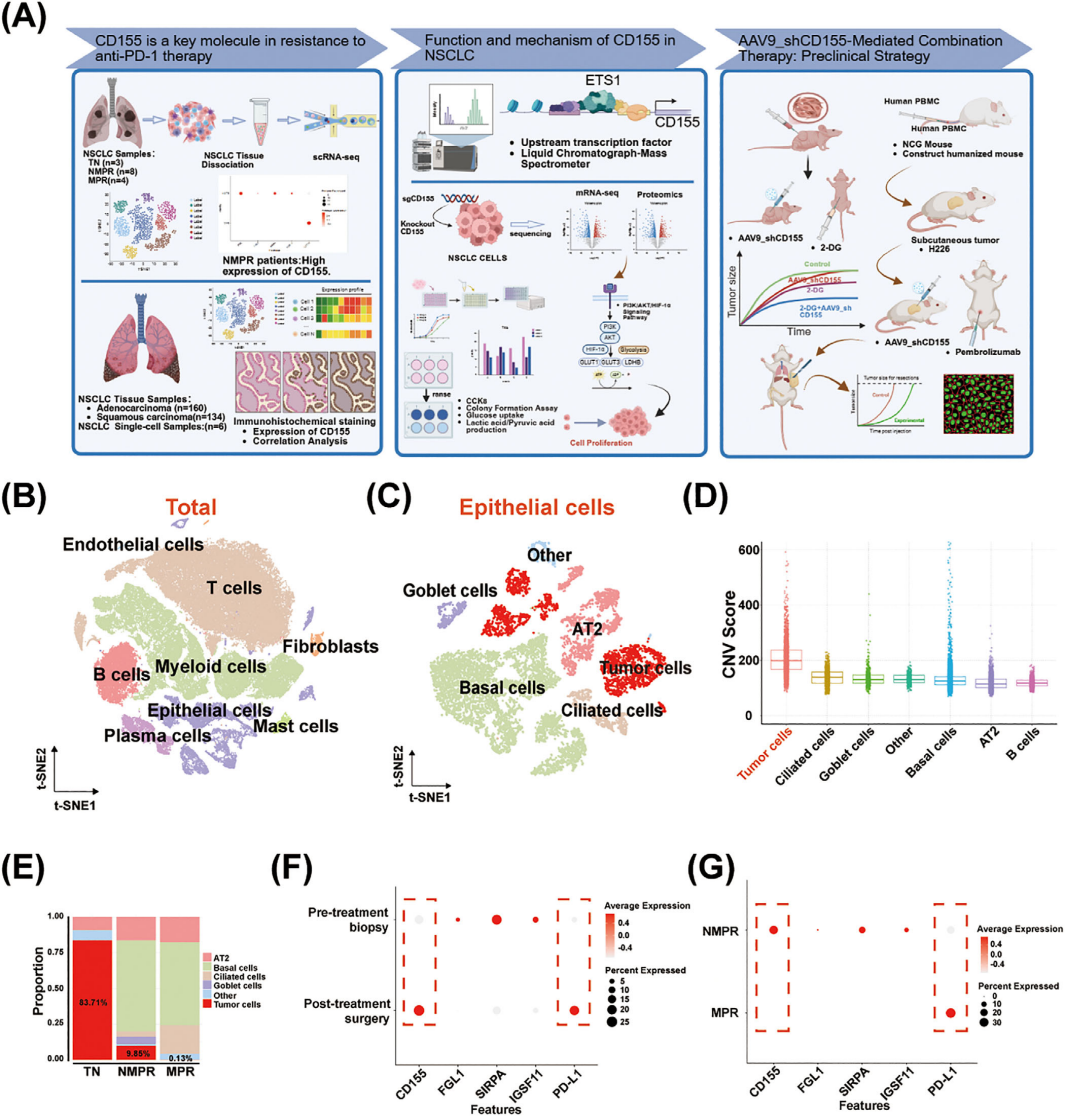
CD155 is a key driver of resistance to anti‐PD‐1 blockade. (A) The strategy diagram of this paper. (B) Approximately 92 330 cells from 15 samples (including 3 TN patients and 12 patients who had undergone neoadjuvant anti‐PD‐1 blockade combined with chemotherapy, which included 4 patients with MPR and 8 patients with NMPR) were defined by specific markers and visualised using a *t*‐distributed Stochastic Neighbor Embedding (*t*‐SNE) plot, where eight colours represented eight cell lineages (data source: Public sc‐RNA‐seq data from GSE207422, sample size *n* = 15; see Section 2 for details). (C) *t*‐SNE plot of epithelial cells, with red representing tumour cells and other colours representing normal epithelial cell subpopulations. (D) Copy number variation score of epithelial cell subpopulations, with tumour cells having the highest malignancy. (E) Proportion of tumour cells among epithelial cells in the TN, NMPR, and MPR patient groups. (F) Bubble plot of immune checkpoint expression in cells treated with anti‐PD‐1 blockade versus untreated cells. (G) Bubble plot of immune checkpoint expression in NMPR and MPR patient groups. Figure 1A was generated using BioRender (https://biorender.com).

Furthermore, our preliminary findings indicate that CD155 is highly expressed in patients with non‐major pathologic response following anti‐PD‐1 therapy. CD155, the poliovirus receptor (PVR or Necl‐5), is a transmembrane glycoprotein.^13^ Under physiological conditions, CD155 is expressed at low levels in endothelial cells, epithelial cells, and other cell types,^14^, ^15^, ^16^, ^17^ yet it is frequently overexpressed across diverse malignancies, including melanoma, pancreatic cancer, cervical cancer, and others.^18^, ^19^, ^20^, ^21^ Its expression is often correlated with unfavourable patient prognosis. CD155 facilitates tumour immune evasion through its interaction with the inhibitory receptor TIGIT on T cells.^19^ Additionally, CD155 expression enhances tumour cell invasiveness and contributes critically to their motility, migration, and proliferative capacity.^22^, ^23^, ^24^ In this study, Figure 1A illustrates the experimental design. We systematically investigated the potential role of CD155 in NSCLC based on scRNA‐seq, RNA‐seq, proteomic, and metabolomic sequencing. We found that CD155 modulates glycolytic metabolism in NSCLC via the PI3K/AKT/HIF‐1α signalling axis, thereby enhancing glucose utilisation and driving malignant progression. Furthermore, concurrent targeting of CD155 and PD‐1 therapies effectively suppressed tumour proliferation and mitigated the limitations of PD‐1 monotherapy, providing new insights for the dual‐targeting strategy against CD155 and PD‐1.

## 2 MATERIALS AND METHODS

### 2.1 Patients and tissue samples

In total, 588 surgical specimens (294 tumours and 294 paired adjacent non‐cancerous tissues) were procured from NSCLC patients undergoing surgical resection at Tangdu Hospital. The inclusion and exclusion criteria were: (1) Confirmed NSCLC diagnosis through pathological biopsy. (2) Complete preoperative laboratory and imaging examination reports. (3) No prior radiotherapy or chemotherapy. (4) Availability of complete follow‐up records. Exclusion criteria: (1) Prior treatment history, such as neoadjuvant therapy, radiotherapy, or chemotherapy. (2) Missing laboratory or imaging examination reports. (3) Absence of follow‐up records. All procedures were approved by the Ethics Committee of Air Force Medical University (No. 202003‐018).

### 2.2 Single‐cell sequencing and analysis

Tumour tissue samples were obtained from 6 NSCLC patients. The tissues were digested into single cells and loaded onto the 10× Genomics platform. scRNA‐seq libraries were constructed using the Chromium Single‐Cell 3′ v3 kit (10× Genomics) according to the manufacturer's protocol. After pooling, the libraries were sequenced on a NovaSeq 6000 (Illumina) with 150‐bp paired‐end reads, and read depth was normalised to cell numbers. Dimensionality reduction, cluster identification, and expression matrix analysis were conducted with the Seurat software (version 3.1.1).

The NSCLC dataset (15 samples) was downloaded from GEO under accession GSE207422 (https://www.ncbi.nlm.nih.gov/geo/query/acc.cgi?acc=GSE207422).^25^ Stringent quality filtering was applied based on four criteria: fewer than 500 expressed genes, a mitochondrial UMI proportion exceeding 20%, a ribosomal UMI ratio above 50%, or a composite housekeeping score (derived from ACTB, GAPDH, and MALAT1) below 1. After QC, 92 330 cells were retained.

### 2.3 Immunohistochemistry (IHC)

A total of 588 tissue samples were prepared as 3‐µm tissue microarrays. Xylene I/II/III, absolute ethanol, as well as 75% and 85% alcohol were used to dewax the tissue microarrays. The microarray chips were placed in citric acid antigen repair buffer and heated for 15 min. To block peroxidase activity, 3% hydrogen peroxide was used for incubation for 20 min. The microarrays were further incubated with antibodies, followed by chromogenic development using a peroxidase substrate. Following this, the nuclei were restained with haematoxylin, and the microarrays were dehydrated and sealed. The antibodies employed are described in Table S1.

Using Aipathwell.v2 software (provided by Wuhan Servicebio Technology Co., China). *H*‐scores were calculated semiquantitatively as: (percentage of weak intensity) × 1 + (percentage of moderate intensity) × 2 + (percentage of strong intensity) × 3.

### 2.4 Transcription factor evaluation

Putative transcription factor binding sites were identified by interrogating two publicly accessible repositories, hTFtarget (https://bio.tools/hTFtarget) and PROMO (https://alggen.lsi.upc.es/cgi‐bin/promo_v3/promo/promoinit.cgi?dirDB=TF_8.3). These binding sites are used to predict potentially valuable TFs.

### 2.5 ChIP‐qPCR and dual luciferase assay

1 × 10^7^ H226 cells were crosslinked, lysed, and sonicated to obtain DNA samples. The DNA was then subjected to immunoprecipitation using a CD155 antibody. A ChIP kit (BersinBio) was employed, and specific primers for the ChIP‐enriched DNA fragments were used for amplification. The binding of ETS1 to the CD155 promoter region was analysed by qPCR.

The CD155‐promoter‐WT plasmid and CD155‐promoter‐MUT plasmid were co‐transfected with the ETS1 plasmid into H226 cells using Hieff TransTM Liposomal Transfection Reagent (YEASEN), respectively. After thoroughly lysing the cells, the Dual Luciferase Reporter Gene Assay Kit (Tsingke Biotechnology) was used to detect the activities of firefly luciferase and Renilla luciferase.

### 2.6 CO‐IP and mass spectrometry

Collect 1 × 10^7^ H226 cells and wash them three times with pre‐cooled PBS. Add 1 mL of cell lysis buffer, and then incubate the lysates with protein‐A/G agarose beads. Pre‐incubate at 4°C for 10–30 min. Divide the cell lysate (containing protease inhibitors) into Input, IP, and IgG groups in a ratio of 1:4:2. Add the CD155 antibody to the IP group and IgG antibody to the IgG group, respectively, and incubate at 4°C with rotation for 12–18 h. Both the groups were washed three times with pre‐cooled washing buffer. The samples were then mixed with loading buffer, heated at 100°C for 10 min, and centrifuged to collect the supernatants. The proteins were then subjected to SDS‐PAGE and visualised using the silver staining method. Mass spectrometry analysis was performed (technology provided by Beijing TsingKe Biotechnology Co., Ltd.).

### 2.7 Cell culture

All the cell lines were obtained from Wuhan Pricella Biotechnology Co., Ltd. (Wuhan, China). Beas‐2B, H226 and H1299 cells were grown in RPMI 1640 (Gibco) supplemented with 10% FBS (OPCEL) at 37°C in a 5% CO_2_ incubator. A549 cells were maintained under identical conditions. Murine LLC cells were cultured in high‐glucose DMEM (Pricella) containing 10% FBS under the same atmospheric conditions.

### 2.8 Knockout of CD155

The sgRNA targeting the human CD155 gene was designed by Genecarer. The ribonucleoprotein (RNP) complex was assembled by mixing chemically synthesised sgRNA with Cas9‐NLS protein at a molar ratio ranging from 5:1 to 10:1, followed by a 10 min incubation at room temperature. A total of 5×10^5^–1×10^6^ A549, H226, or H1299 cells were trypsinised, resuspended, combined with the RNP complex, and electroporated using the Neon Transfection System (Thermo Fisher) according to the manufacturer's recommendations. After electroporation, cells were seeded into 24‐well plates and cultured for 72 h. Genomic DNA was then extracted via cell lysis, and the target region was amplified by PCR with specific primers. These amplicons were then sent for sequencing to evaluate editing efficiency. To establish monoclonal cell lines, the edited cells were diluted to a concentration of one cell per 100 µL and seeded into 96‐well plates. After 2–3 weeks of culture, single clones were picked, expanded, and validated for CD155 knockout by Sanger sequencing and Western blot. This experimental procedure was provided by Genecarer.

### 2.9 Western blot

Total protein was extracted using a protein collection kit (Invent Biotechnologies). Samples were spun at 12 000 rpm (5 min, 4°C), and supernatants were collected. Equivalent protein aliquots underwent 10% SDS‐PAGE and electrotransfer onto nitrocellulose. Membranes were blocked with QuickBlock (Beyotime) for 15 min, exposed to primary antibodies overnight at 4°C, and subsequently incubated with secondary antibodies. The relative protein levels were analysed using ImageJ software. The antibodies employed are described in Table S1.

### 2.10 CCK‐8

1 × 10^3^ cells per well were dispensed into 96‐well plates and examined at 24 h intervals with the CCK‐8 kit (Saint‐Bio). After 48 h, the medium in the 2‑deoxy‑D‑glucose (2‑DG) group (TargetMol) was replaced with medium containing 9 mM 2‑DG, and the cells were further cultured for 72, 96, and 120 h. Briefly, 10 µL of CCK‐8 reagent was combined with 100 µL of Gibco RPMI 1640 medium containing 1000 cells. The reaction was incubated for 1–4 h, and the optical density was recorded at 450 nm.

### 2.11 Colony formation assay

3 × 10^3^ cells from both the negative control (NC) and CD155 knockout (KO) groups of NSCLC cell lines were seeded in a 6‐well plate. After 4 days, the medium in the 2‐DG group was replaced with fresh medium containing 9 mM 2‐DG. After 10 days, the cells were fixed with 4% formaldehyde, stained with crystal violet, washed, and air‐dried for imaging.

### 2.12 Transwell assay

To the lower chambers of 24‐well plates, 600 µL of RPMI 1640 containing 20% FBS (with 30 µL of 180 mM 2‐DG added for the 2‐DG group) was dispensed. A total of 5×10^4^ NSCLC cells from NC and CD155‐KO groups were placed into the upper Transwell chambers in serum‐free RPMI 1640, adjusting the volume to 200 µL (with 10 µL of 180 mM 2‐DG was added for the 2‐DG group). The chambers were then placed into the 24‐well plate and incubated for 36–48 h. After incubation, the chambers were removed, and cells were fixed (4% paraformaldehyde) and stained (2.5% crystal violet). Cells that had migrated to the lower surface were quantified using ImageJ software.

### 2.13 Scratch wound healing assay

NC and CD155‐KO groups of NSCLC cell lines were seeded into 6‐well plates, ensuring consistent cell numbers (with a growth density of 80%–90%). Using a 200 µL pipette tip, a linear wound was generated across each well, and detached cells were removed by three PBS washes. Then, 10 mL of culture medium was added (the 2‐DG group used culture medium containing 9 mM 2‐DG). Wound healing was tracked and photographed at 0, 24, and 48 h post‐scratch. The assay was performed in triplicate.

### 2.14 mRNA‐sequencing, proteomic, and differential expression analysis

Total RNA was extracted from A549 cells (1×10^7^ cells) using TRIzol (Mei5bio). Additionally, total protein was extracted from 1×10^7^ A549 cells. RNA‐sequencing and proteomic analyses were performed on the RNA and protein samples, respectively. The enrichment pathways of differentially expressed genes and proteins were assessed using Kyoto Encyclopedia of Genes and Genomes (KEGG), Gene Ontology (GO), and Gene Set Enrichment Analysis (GSEA). The sequencing and analysis of A549 cells were provided by Tsingke Biotechnology Co., Ltd.

### 2.15 Metabonomic analysis

A549 and A549_CD155‐KO cells (1×10^7^ cells each) were seeded in T75 flasks. After adherence, cells were trypsinised, and spun at 1000 rpm (5 min). For metabolite extraction, 250 µL of ultrapure water was added to the cell pellet, followed by vortexing for 30 s and cell lysis for 15 min. A 50 µL portion of the lysate was set aside for BCA protein quantification. To the remaining 200 µL, 800 µL of pre‐cooled methanol‐acetonitrile (1:1) was added, followed by vortexing, sonication in an ice‐water bath, incubation at –40°C (1 h), and centrifugation at 12 000 rpm (15 min, 4°C). Equal volumes of supernatant from each sample were pooled and dried under vacuum. The dried residue was taken up in a reconstitution solvent (methanol:acetonitrile:water = 2:2:1) spiked with isotope‐labelled internal standards. After vortex‐mixing (30 s) and ice‐water sonication (10 min), the mixture was centrifuged (12 000 rpm, 15 min, 4°C). The clarified supernatant was then analysed by LC‐MS/MS. A pooled QC sample was prepared by combining equal volumes of each supernatant and was analysed in parallel with the test samples.

LC‐MS/MS separation was carried out on a Thermo Vanquish UHPLC system equipped with a Waters ACQUITY UPLC BEH Amide column. Mobile phase A consisted of 25 mmol/L ammonium acetate and 25 mmol/L ammonia in water, and mobile phase B was acetonitrile. The autosampler temperature was maintained at 4°C, and a 2 µL injection volume was used. Mass spectrometric detection was conducted on an Orbitrap Exploris 120 mass spectrometer operating in both positive and negative ionisation modes. Both full scan MS and data‐dependent MS/MS spectra were acquired. All mass spectrometry parameters were optimised to ensure high sensitivity and resolution. The sequencing and analysis were provided by Tsingke Biotechnology Co., Ltd.

### 2.16 Glucose uptake, lactate production, and pyruvate production assays

Cells were plated in 24‐well plates at a density of 3 × 10^5^ cells per well in 300 µL of medium. After 24 h, the culture supernatants were harvested and centrifuged at 3000 rpm for 10 min. Concentrations of glucose, lactate, and pyruvate were determined using assay kits (Nanjing Jiancheng) per the manufacturers' instructions.

### 2.17 Isolation of peripheral blood mononuclear cells (PBMCs)

Whole blood was diluted with an equal volume of PBS, thoroughly mixed, and then allowed to sit undisturbed. A 15 mL centrifuge tube was charged with 4 mL of lymphocyte separation medium (TBD Science), onto which 4 mL of diluted whole blood was gently layered (diluted blood: lymphocyte separation medium = 1:1; this step was performed slowly to prevent mixing of the blood and separation medium). The centrifuge tube was placed in a centrifuge and spun at 400 × *g* for 35 min at room temperature (with a natural deceleration to a stop). Following centrifugation, the buffy coat was transferred to a fresh tube, rinsed with PBS to eliminate residual separation medium, and pelleted by centrifugation at 350 × *g* for 7 min at 4°C. After discarding the supernatant, the wash step was repeated once. The resulting PBMCs pellet was resuspended in PBS and counted. This study was approved by the Ethics Committee of Air Force Medical University (No. GKJ‐Y‐202403‐074).

### 2.18 Tumour formation assay in nude mice

Male BALB/cJGpt‐Foxn1nu/Gpt mice (5 weeks old, 20–25 g; GemPharmatech, Beijing) were used. Animal experiments were approved by the Ethics Committee of Air Force Medical University (No. 20240126). Mice were randomly assigned to two groups: NC and CD155_KO. Each mouse received a subcutaneous injection of 8 × 10^6^ A549_NC or A549_CD155_KO cells into the axillary region. Tumour‐bearing mice were weighed and tumour dimensions were recorded every 2–4 days. Tumour volumes (mm^3^) were calculated using the formula: length × width^2^/2.

### 2.19 AAV9_shCD155 treatment model

Male BALB/cJGpt‑Foxn1nu/Gpt mice (5 weeks old, 20–25 g) from GemPharmatech (Beijing, China) were used. Subcutaneously injected (right axilla) 8 × 10^6^ A549 cells into the mice, and randomly assigned the mice into two groups (*n* = 5). When the tumour reached a volume of 100–200 mm^3^, 40 µL of HBAAV2/9‐h‐CD155 shRNA‐EGFP (1.2×10^12^ vg/mL, synthesised by HanBio Therapeutics, China) was directly locally injected into multiple parts of the tumour. After 35 days, the tumour growth rates were compared between the NC group (untreated) and the AAV9_shCD155 group. When the control group tumour volume reached 1000 mm^3^, the tumour was surgically removed, and 50 mm^3^ of tumour tissue was re‐implanted under the skin of the mice. Tumour recurrence was compared between the two groups of mice.

Subcutaneous tumours were established in 20 male mice (BALB/cJGpt‐Foxn1nu/Gpt; 5 weeks old; 20–25 g) by subcutaneously inoculating 5×10^5^ LLC cells. Four days later, mice were randomly assigned to four groups. The treatment group received local injections of AAV9_shCD155 (20 µL every 4 days) and intraperitoneal injections of 2‐DG (1800 mg/kg every 2 days). After 20 days, the tumour sizes were compared to evaluate the therapeutic efficacy.

### 2.20 Humanised mouse model

To enable evaluation of human immune checkpoint blockade therapies in vivo, a humanised mouse model was established by reconstituting immunodeficient NCG mice with human PBMCs. NCG mice (NOD/ShiLtJGpt‐*Prkdcem26Il2rgem26*/Gpt) lack functional T, B, and NK cells, making them highly permissive for human cell engraftment. Human PBMCs were purified from healthy donor blood by density gradient centrifugation. Each NCG mouse (*n* = 12) received an intravenous (tail vein) injection of 2 × 10^7^ cells suspended in PBS. Two weeks post‐injection, peripheral blood was collected from each mouse, and engrafted human T cells were identified via flow cytometry using anti‐CD45 and anti‐CD3 antibodies. Mice were considered successfully humanised if human CD45^+^CD3^+^ T cells constituted ≥25% of peripheral blood mononuclear cells, and were subsequently used for tumour implantation and treatment.^26^, ^27^

### 2.21 Establishment of immune‐CDX models

Twelve humanised mice were subcutaneously injected with H226 cells (1 × 10^7^ cells per mouse) into the right axilla. Drug treatment was initiated when tumour volumes reached 50–120 mm^3^, and body weight and tumour growth were recorded every 2 to 4 days. Mice were allocated to four groups: Control group: no treatment; AAV9‐shCD155 group: intratumourally inject 40 µL of HBAAV2/9‐h‐CD155 shRNA‐EGFP at multiple sites; Pembrolizumab group: pembrolizumab was administered intraperitoneally at 10 mg/kg once tumours reached 100–200 mm^3^; AAV9‐shCD155 + Pembrolizumab group. On day 21, mice were sacrificed and tumours excised. Tumour sizes were compared among the four groups, and growth curves were plotted.

### 2.22 Immunofluorescence staining

The tissue sections were dewaxed and rehydrated by sequential treatment with xylene (10 min, 10 min, and then 5 min), followed by immersion in absolute ethanol (5 min and then another 5 min), 95% ethanol (5 min), and 85% ethanol (5 min), and finally rinsed with distilled water. For antigen retrieval, the sections were placed in antigen retrieval buffer, heated appropriately, and allowed to cool naturally. Sections were encircled with a hydrophobic pen, and non‐specific binding was blocked by incubation with BSA for 30 min at room temperature. Primary antibodies were prepared according to the manufacturer's instructions. Sections were incubated with the primary antibody cocktail overnight at 4°C, followed by three PBS washes (5 min each) and incubation with the corresponding secondary antibody for 50 min at room temperature in the dark. For nuclear counterstaining, sections were incubated with DAPI for 10 min at room temperature in the dark, washed three more times with PBS (5 min each), mounted with anti‐fade medium, and imaged. Technical support was provided by Servicebio. Details of the antibodies used are provided in Table S1.

### 2.23 Statistical analysis

GraphPad Prism 10.1.2 was used for all statistical calculations. Quantitative data are presented as means ± SEM. Group comparisons were performed using Student's *t*‐test for sample sizes under 30, while the Mann–Whitney *U* test was applied to larger cohorts (*n* > 60) with non‐Gaussian distributions. *p*‐value of less than .05 was considered statistically significant.

## 3 RESULTS

### 3.1 CD155 is a key molecule for resistance to anti‐PD‐1 therapy

Based on 15 external single‐cell samples of NSCLC (the sources of single‐cell Sequencing data:GSE207422^25^), including 3 from treatment‐naive patients (TN) and 12 from patients who had undergone neoadjuvant anti‐PD‐1 blockade combined with chemotherapy (including 4 patients with major pathological response [MPR]) and 8 patients with non‐major pathological response [NMPR]), we classified approximately 92 330 single cells into 8 subgroups using specific markers. These subgroups included endothelial cells, T cells, plasma cells, myeloid cells, mast cells, fibroblasts, epithelial cells, and B cells. Given the epithelial origin of lung cancer, we focused on the epithelial cells (Figures 1B and S1A). By re‐clustering the epithelial cells, we divided them into 25 clusters and defined them into six cell populations based on specific markers and Infer Copy Number Variation (InferCNV): tumour cells, goblet cells, basal cells, ciliated cells, AT2, and others (Figures 1C and S1B–D). Malignancy was assessed in epithelial subgroup cells using InferCNV analysis, which revealed a high CNV score in tumour cells (Figure 1D). The proportion of tumour cells was also evaluated across TN, MPR, and NMPR patient groups. Research findings indicate that MPR patients showed a lower proportion of tumour cells than TN patients (.13% vs. 83.71%) (Figure 1E). To identify key molecules potentially associated with resistance in patients undergoing neoadjuvant therapy, we examined changes in immune checkpoints expression (CD155, FGL1, SIRPA, IGSF11, PD‐L1) on tumour cells. Comparisons were made between pre‐treatment biopsy samples from patients who had not received PD‐1 blockade and post‐treatment surgical specimens from those who had, CD155 and Programmed Death‐Ligand 1(PD‐L1) expression were upregulated after treatment (Figure 1F). Furthermore, we compared the expression levels of CD155 and PD‐L1 between the NMPR and MPR patient groups after treatment. Notably, we observed that CD155 expression was markedly elevated in NMPR patients, whereas PD‐L1 expression remained relatively low (Figure 1G), suggesting a potential compensatory relationship that may promote resistance to anti‐PD‐1 therapy. However, the underlying mechanism requires experimental validation.

### 3.2 CD155 is overexpressed in NSCLC and transcriptionally regulated by ETS1

**FIGURE 2:**
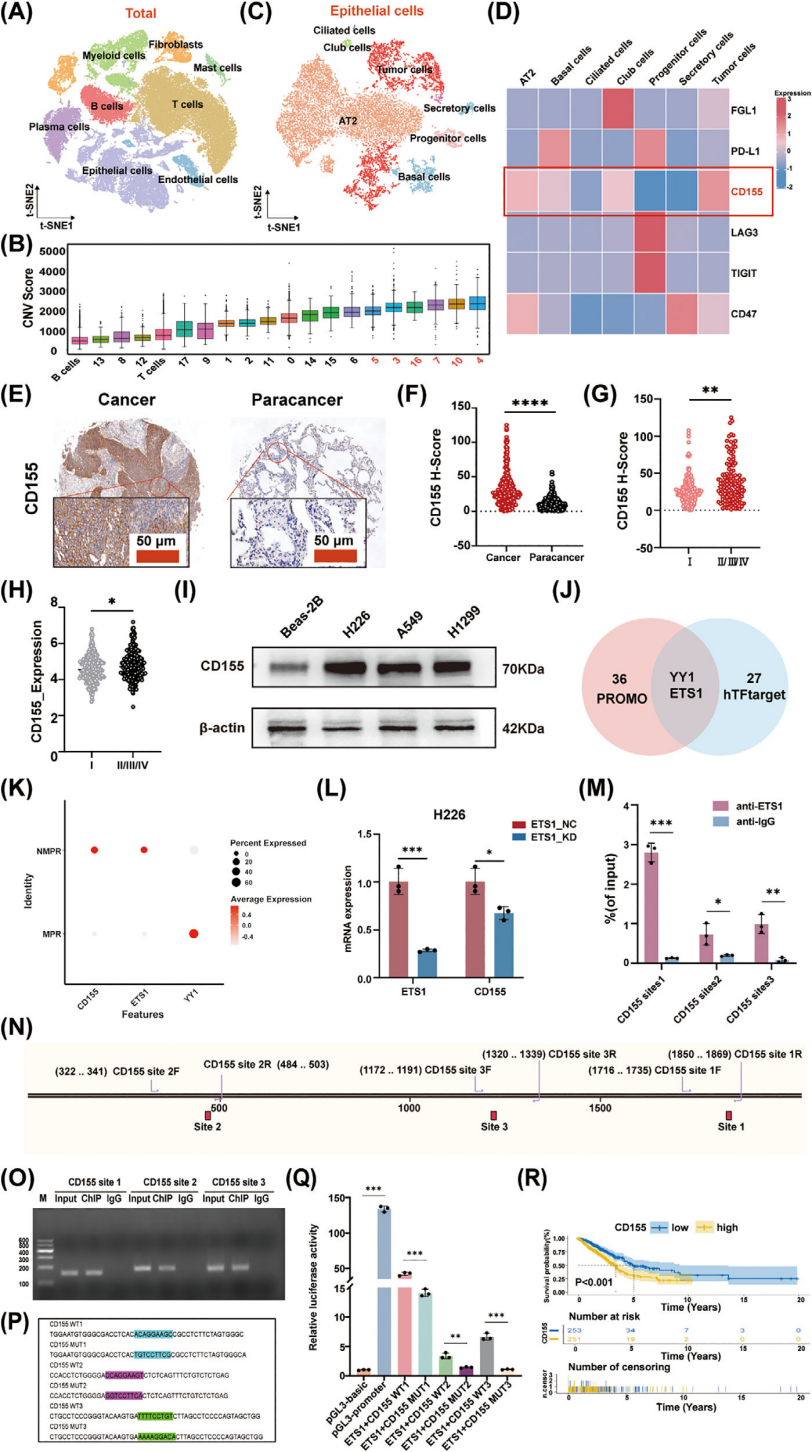
CD155 is transcriptionally regulated by ETS1 and associated with adverse patient outcomes. (A) *t*‐SNE visualisation demonstrating cellular composition across six samples. (B, C) CNV‐based malignancy scores and *t*‐SNE projection of epithelial cells, with tumour cells represented in red. (D) Heatmap assessment of CD155 expression in epithelial cells. (E, F) tissue microarray was constructed from 294 NSCLC tissue specimens, and CD155 staining was conducted using IHC. (G, H) Assessment of CD155 expression in early‐stage and mid‐to‐late stage NSCLC by IHC and TCGA database analysis. (I) Western blot detection of CD155 protein expression levels in Beas‐2B and NSCLC cell lines (H1299, A549, H226), using Beas‐2B as an internal reference. (J) Upstream transcription factors regulating CD155, such as ETS1 and YY1, were predicted via the PROMO and hTFtarget databases. (K) A comparison of ETS1 and YY1 expression levels in epithelial tumour cells was conducted between NMPR and MPR patients. (L) In H226 cells, ETS1 was knocked down by siRNA, and the mRNA expression of CD155 was evaluated. (M–Q) The binding site of ETS1 on CD155 was identified via ChIP‐qPCR and dual‐luciferase reporter assays. (R) Evaluation of survival probability and number at risk associated with CD155 in patients from the TCGA database. In F, G, and H, data are expressed as mean ± SEM, and statistical significance was determined using the Wilcoxon rank‐sum test. In L, M, and Q, data are presented as mean ± SEM and statistical significance was analysed using the an unpaired *t*‐test. *p < .05, **p < .01, ***p < .001, ****p < .0001.

To further validate the expression pattern of CD155, we subjected six additional NSCLC samples to scRNA‐seq. Cells were annotated as T cells, myeloid cells, epithelial cells, B cells, endothelial cells, fibroblasts, mast cells, and plasma cells (Figures 2A and S2A–K). Within the epithelial compartment, 17 distinct clusters were identified. Based on copy number variation (CNV) analysis, these clusters were stratified into malignant tumour cells and non‐malignant epithelial cells, including AT2, basal cells, club cells, ciliated cells, secretory cells, and progenitor cells (Figures 2B and C and S2L). Subsequent analysis showed that CD155 was significantly upregulated in tumour cells relative to all other epithelial cell subtypes (Figure 2D).

CD155 expression was additionally assessed by immunohistochemistry (IHC) in 294 primary NSCLC samples and an equal number of adjacent non‐cancerous tissue samples (Figure 2E). The expression of CD155 was significantly higher in NSCLC specimens than in adjacent normal tissues (*p *< .0001; Figure 2F). Furthermore, the expression of CD155 in early‐stage NSCLC was lower than that in mid‐to‐late stage NSCLC (*p *< .01; Figure 2G), a trend also observed in The Cancer Genome Atlas (TCGA) data (*p* < .05; Figure 2H). Subsequently, in vitro assessment confirmed higher CD155 protein levels in NSCLC cell lines (H1299, A549, H226) than in normal bronchial epithelial cells (Beas‐2B) (Figure 2I).

To investigate the mechanism underlying CD155 upregulation, we explored its transcriptional regulation.​ Bioinformatic prediction using the PROMO and hTFtarget databases identified potential upstream transcription factors, including E26 transformation‐specific transcription factor 1(ETS1) and Yin Yang 1(YY1) (Figure 2J). Analysis of our scRNA‐seq data showed that ETS1 expression was markedly elevated in epithelial tumour cells from NMPR patients relative to those from MPR patients​ (Figure 2K), suggesting a potential link to therapy resistance. Functional validation in H226 cells demonstrated that siRNA‐mediated knockdown of ETS1 significantly reduced CD155 mRNA expression​ (*p* < .05; Figure 2L). To delineate the precise binding regions of ETS1 within the CD155 promoter, we performed chromatin immunoprecipitation quantitative PCR (ChIP‐qPCR), which identified three specific ETS1‐binding regions within the CD155 promoter (Figure 2M–O). The functional significance of these sites was validated by dual‐luciferase reporter assays. Versus the wild‐type promoter, mutation of any of the three predicted ETS1‐binding sites markedly impaired ETS1‐driven transcriptional activation of the CD155 promoter, and the most pronounced reduction was observed with site 1 mutation (*p* < .001; Figure 2P and Q). These results establish that ETS1 directly targets the CD155 promoter and activates its transcription.

Analysis of TCGA data revealed that high CD155 expression correlated with significantly shorter overall survival in NSCLC patients (*p* < .001), indicating that CD155 may serve as an independent prognostic indicator of poor outcomes (Figure 2R).

### 3.3 Multi‐omics investigation of the potential functions of CD155 in NSCLC

**FIGURE 3:**
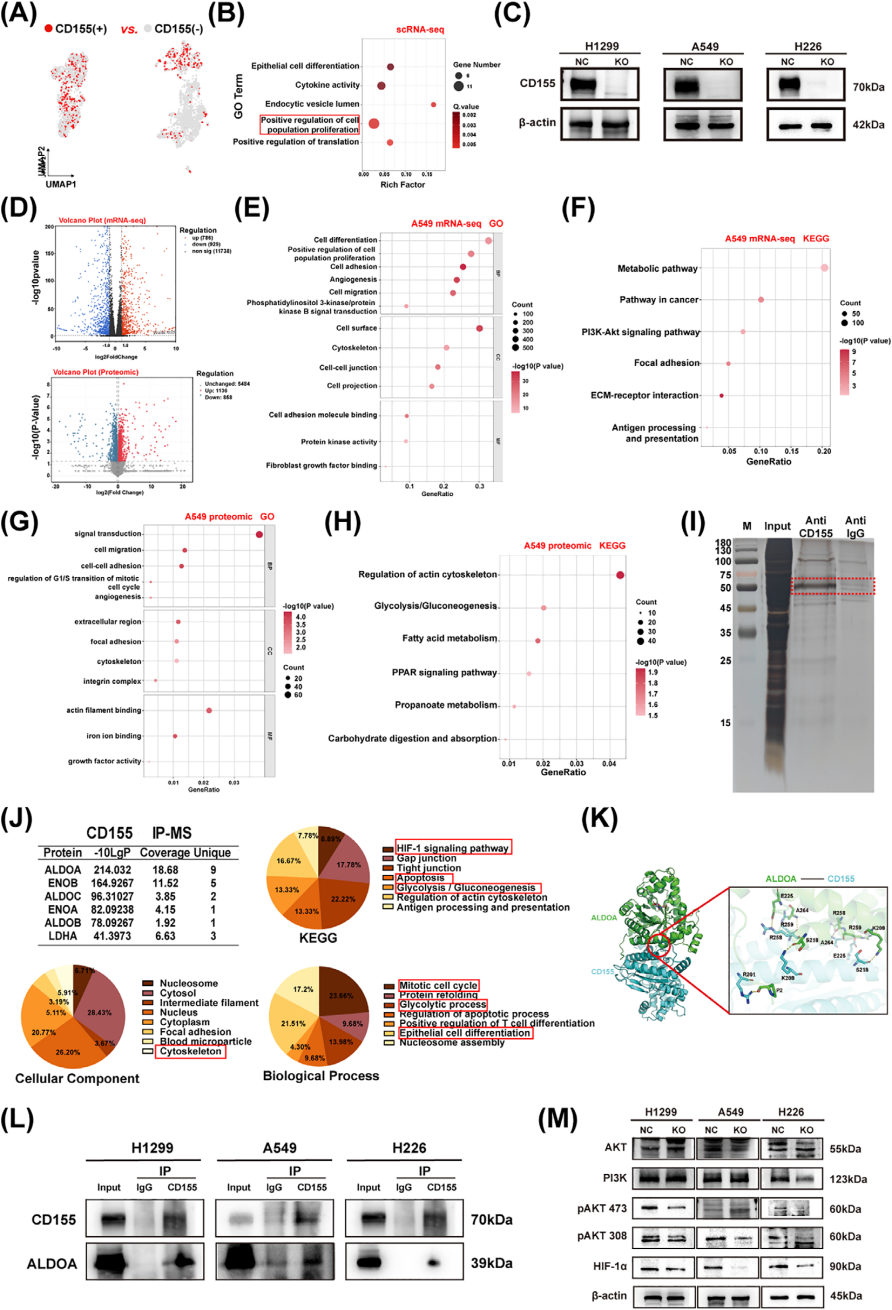
CD155 regulates gene expression, interacts with ALDOA, and activates the PI3K/AKT/HIF‐1α axis in NSCLC. (A) Differentially expressed genes between the CD155(+) and CD155(–) groups were identified, and (B) GO enrichment analysis was performed. (C) Western blot analysis confirming CD155 knockout in NSCLC cell lines. (D) Volcano plots displaying Differentially Expressed Genes (DEGs) (mRNA‐seq) and Differentially Expressed Proteins (DEPs) (proteomics) identified in A549 cells upon CD155 knockout. (E, F) Bubble plots of GO and KEGG enrichment analyses for transcriptomic data. (G, H) Bubble plots of GO and KEGG enrichment analyses for proteomic data. (I, J) CD155‐associated proteins were identified by co‐immunoprecipitation and mass spectrometry. (K) Molecular interaction model depicting the predicted binding interface between CD155 (blue) and ALDOA (green). (L) Co‐IP assay validating the protein‐protein interaction between CD155 and ALDOA.(M) Western blot analysis showing that CD155 knockout inhibits the PI3K/AKT/HIF‐1α signalling pathway.

To investigate the molecular mechanisms underlying CD155 function in NSCLC, we performed multi‐omics profiling at the transcriptomic (scRNA‐seq and bulk RNA‐seq) and proteomic levels. First, based on the tumour cell populations identified in Figure 2C, we stratified these malignant cells according to CD155 expression into CD155(+) and CD155(–) subgroups, and then compared their transcriptomic profiles using scRNA‐seq data (Figure 3A). To explore the functional relevance of CD155 in NSCLC, Gene Ontology (GO) enrichment analysis was applied to the genes differentially expressed between CD155(+) and CD155(–) tumour cells at the single‐cell level. These genes were significantly enriched in biological processes related to cell proliferation (Figure 3B). This finding suggests that CD155 overexpression may actively promote malignant progression in NSCLC by enhancing tumour cell proliferation.

To functionally validate the role of CD155, CD155 knockout (KO) cell lines were generated in H1299, A549, and H226 NSCLC cells using the CRISPR‑Cas9 system. Successful knockout efficiency was confirmed by Western blot analysis (Figure 3C). Subsequent multi‐omics analysis of A549_CD155‐KO cells revealed extensive molecular alterations: 786 genes were upregulated and 929 downregulated at the transcriptomic level, while 1136 proteins were upregulated and 858 downregulated at the proteomic level (Figure 3D). Transcriptomic GO and Kyoto Encyclopedia of Genes and Genomes (KEGG) analyses revealed significant enrichment of differentially expressed genes in cancer‐related processes and pathways, including cell proliferation, adhesion, migration, apoptosis, and the PI3K/AKT pathway (Figure 3E and F). Similarly, proteomic GO and KEGG analyses revealed that the differentially expressed proteins were markedly enriched in processes including cell migration, cell‐cell adhesion, cell cycle, apoptosis, and tumour glycolysis (Figure 3G and H).

**FIGURE 4:**
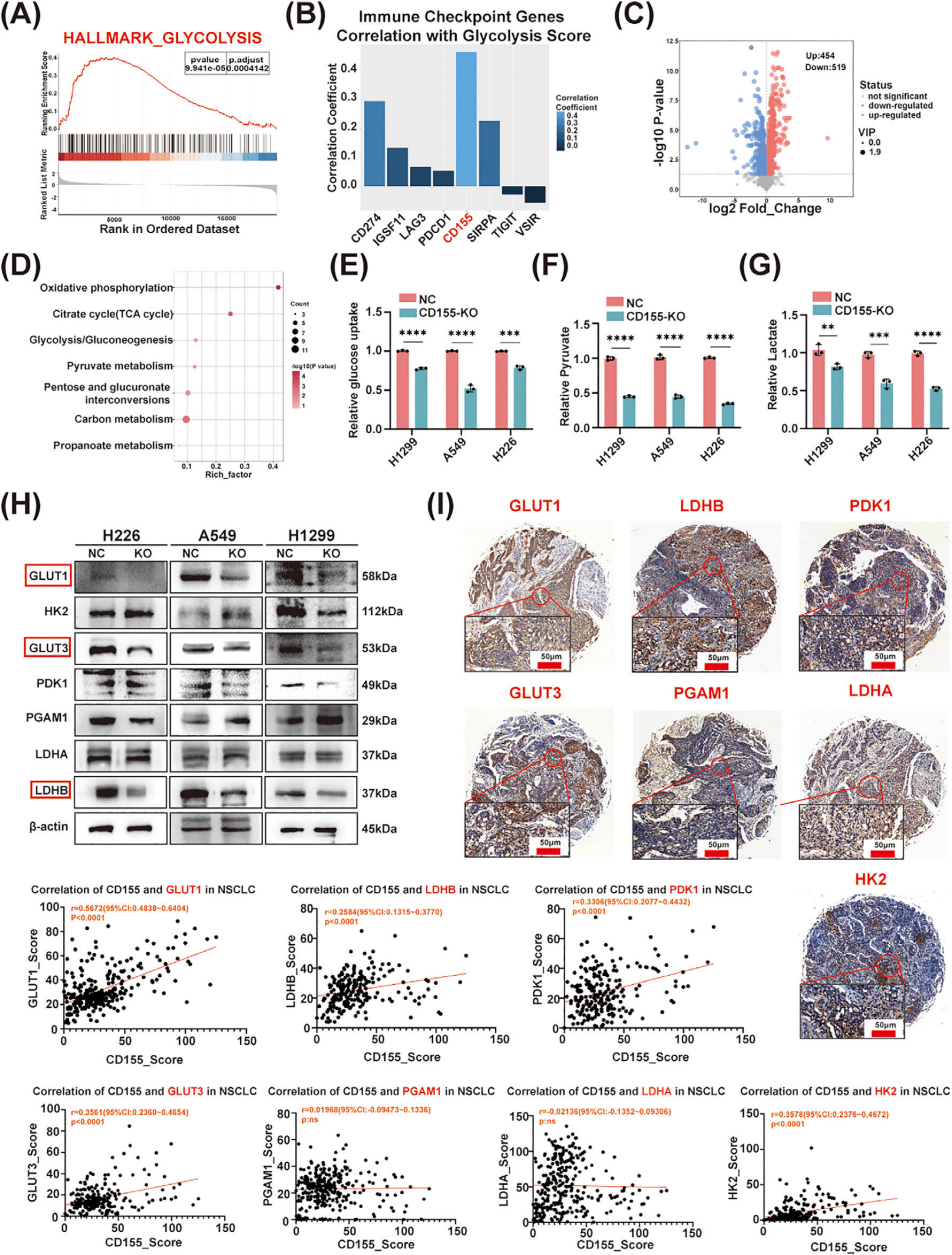
CD155 reshapes the glucose metabolism in NSCLC. (A) GSEA revealed that CD155 was significantly enriched in the glycolysis pathway in TCGA data. (B) The correlation coefficients between immune checkpoint genes and glycolysis were assessed. (C) Volcano plot displaying differentially abundant metabolites from metabolomic sequencing of A549_CD155‐knockout cells compared to control cells. Metabolites increased and decreased in abundance are coloured blue and red, respectively. (D) Bubble plot of KEGG enrichment analysis for the differential metabolites. (E–G) Assessment of the effects of CD155 knockout on glucose uptake, lactate production, and pyruvate production in H1299, A549, and H226 cell lines. (H) Western blotting validated the expression levels of glycolysis‐related proteins following CD155 knockout. (I) Immunohistochemistry was employed to examine the association between CD155 and glycolysis‐related proteins. In E, F, and G, data are presented as mean ± SEM and statistical significance was analysed using the an unpaired *t*‐test. **p < .01, ***p < .001, ****p < .0001.

To identify the direct protein interactors that mediate CD155's functions, we performed co‐immunoprecipitation (Co‐IP) followed by liquid chromatography‐tandem mass spectrometry (LC‐MS/MS) in H226 cells (Figure 3I). The mass spectrometry data revealed that CD155‐associated proteins were significantly enriched in glycolysis‐related pathways. Notably, CD155 appeared to interact with several glycolytic enzymes, thereby potentially influencing key biological processes such as cytoskeleton assembly, epithelial cell differentiation, and apoptosis, all of which are closely linked to tumour malignancy (Figure 3J). Among the putative interactors, aldolase A (ALDOA), a rate‐limiting enzyme in glycolysis, was identified as a novel candidate binding partner of CD155 based on its high peptide coverage and binding affinity. To further validate this interaction, we performed molecular docking simulations, which predicted a potential binding interface between CD155 and ALDOA. Specifically, positively charged basic residues on CD155 – including Arg258, Arg259, Lys208, and Arg201 – were found to form hydrogen bonds with residues on the surface of ALDOA, such as Glu225, Ser218, and Ala264, representing the primary interaction hotspots (Figure 3K). An independent co‐immunoprecipitation assay subsequently verified the direct physical interaction between CD155 and ALDOA (Figure 3L). Finally, to explore the upstream signalling mechanisms by which CD155 regulates glycolysis, we examined the PI3K/AKT/HIF‐1α pathway. Western blot analysis demonstrated that CD155 knockout markedly reduced the phosphorylation of AKT (p‐AKT‐Thr308) and suppressed the expression of its downstream effector HIF‐1α, indicating that CD155 promotes glycolysis at least in part through activation of the PI3K/AKT/HIF‐1α axis (Figure 3M). To further explore the relationship between CD155 and glycolysis, we performed Gene Set Enrichment Analysis (GSEA) on the mRNA expression profiles of NSCLC patients from the TCGA database. Consistent with our mass spectrometry findings, glycolysis activity was significantly associated with CD155 expression (*p* = .0004142) (Figure 4A and B). We then directly assessed the metabolic consequences of CD155 loss by conducting untargeted metabolomic analysis on CD155‐knockout A549 cells. Compared with control cells, CD155‐KO cells exhibited widespread metabolic alterations, with 454 metabolites significantly increased and 519 metabolites decreased (Figure 4C). KEGG pathway enrichment analysis of the differentially abundant metabolites revealed significant disruptions in the TCA cycle, glycolysis/gluconeogenesis, and pyruvate metabolism (Figure 4D).

To functionally validate the impact of CD155 on glycolytic flux, we measured key metabolic parameters in three NSCLC cell lines (H1299, A549, and H226) following CD155 knockout. Across all three lines, CD155 deletion consistently led to significantly impaired glucose uptake, reduced lactate production, and decreased pyruvate production compared to control cells (*p *< .01, Figure 4E–G). These findings demonstrate that CD155 is essential for sustaining the glycolytic capacity of NSCLC cells. Accordingly, Western blotting revealed that CD155 knockout diminished the protein levels of key glycolytic enzymes, including GLUT1, GLUT3, and LDHB (Figure 4H). Overall, these findings demonstrate that CD155 facilitates glycolysis in NSCLC by upregulating multiple rate‐limiting enzymes and transporters.

IHC was performed to examine the relationship between CD155 and glycolysis‐related proteins in NSCLC, which revealed a positive association with GLUT1 (*r* = .5672, *p *< .0001), LDHB (*r* = .2584, *p *< .0001), PDK1 (*r* = .3306, *p *< .0001), GLUT3 (*r* = .3561, *p *< .0001), and HK2 (*r* = .3578, *p *< .0001) (Figure 4I).

### 3.4 CD155 promotes tumour malignant progression through the glycolysis

**FIGURE 5:**
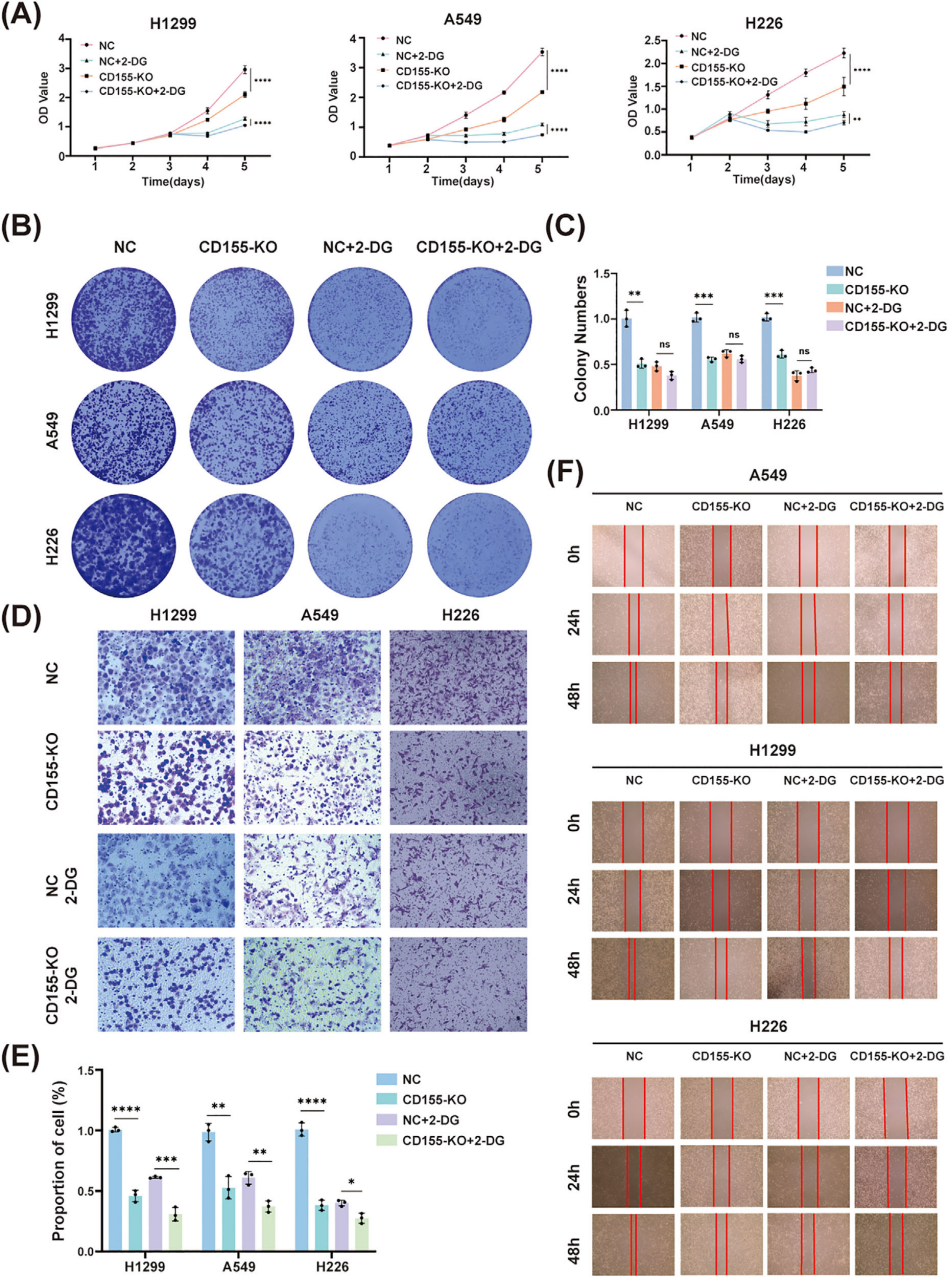
CD155 promotes the proliferation and metastatic phenotype of NSCLC cells via the glycolysis pathway. (A–C) Using CCK‐8 and colony formation assays, we compared the proliferation of CD155‐knockout (KO) and negative control (NC) A549, H1299, and H226 cells under both normal and glycolysis‐inhibited (2‐DG) conditions. (D‐F) The migration ability of NSCLC cells after CD155 knockout was confirmed by Transwell assay and wound‐healing assay. In A, C, and E, data are presented as mean ± SEM and statistical significance was analysed using the an unpaired *t*‐test. ns: not significant, *p < .05, ** *p* < .01, *** *p* < .001, **** *p* < .0001.

We next investigated whether CD155 promotes the malignant progression of NSCLC through glycolysis. To test this hypothesis, we treated control and CD155‐knockout (KO) NSCLC cell lines with the glycolysis inhibitor 2‐deoxy‐D‐glucose (2‐DG) to create a glycolysis‐suppressed microenvironment. Under normal culture conditions, CD155 KO significantly reduced cell proliferation, as measured by Cell Counting Kit‐8(CCK‐8) and colony formation assays. Notably, when glycolysis was inhibited by 2‐DG, the antiproliferative effect of CD155 KO was markedly attenuated, suggesting that the growth advantage conferred by CD155 largely depends on intact glycolytic flux (Figure 5A–C). Similarly, CD155 KO substantially impaired the migratory capacity of NSCLC cells under normal conditions. However, in the presence of 2‐DG, the difference in migration between control and CD155‐KO cells was much less pronounced, indicating that CD155 promotes cell migration primarily through glycolysis‐dependent mechanisms (Figure 5D–F). Collectively, these results support the model that CD155 drives NSCLC proliferation and migration by enhancing glycolysis, and that disrupting glycolysis largely abrogates the pro‐malignant functions of CD155.

**FIGURE 6:**
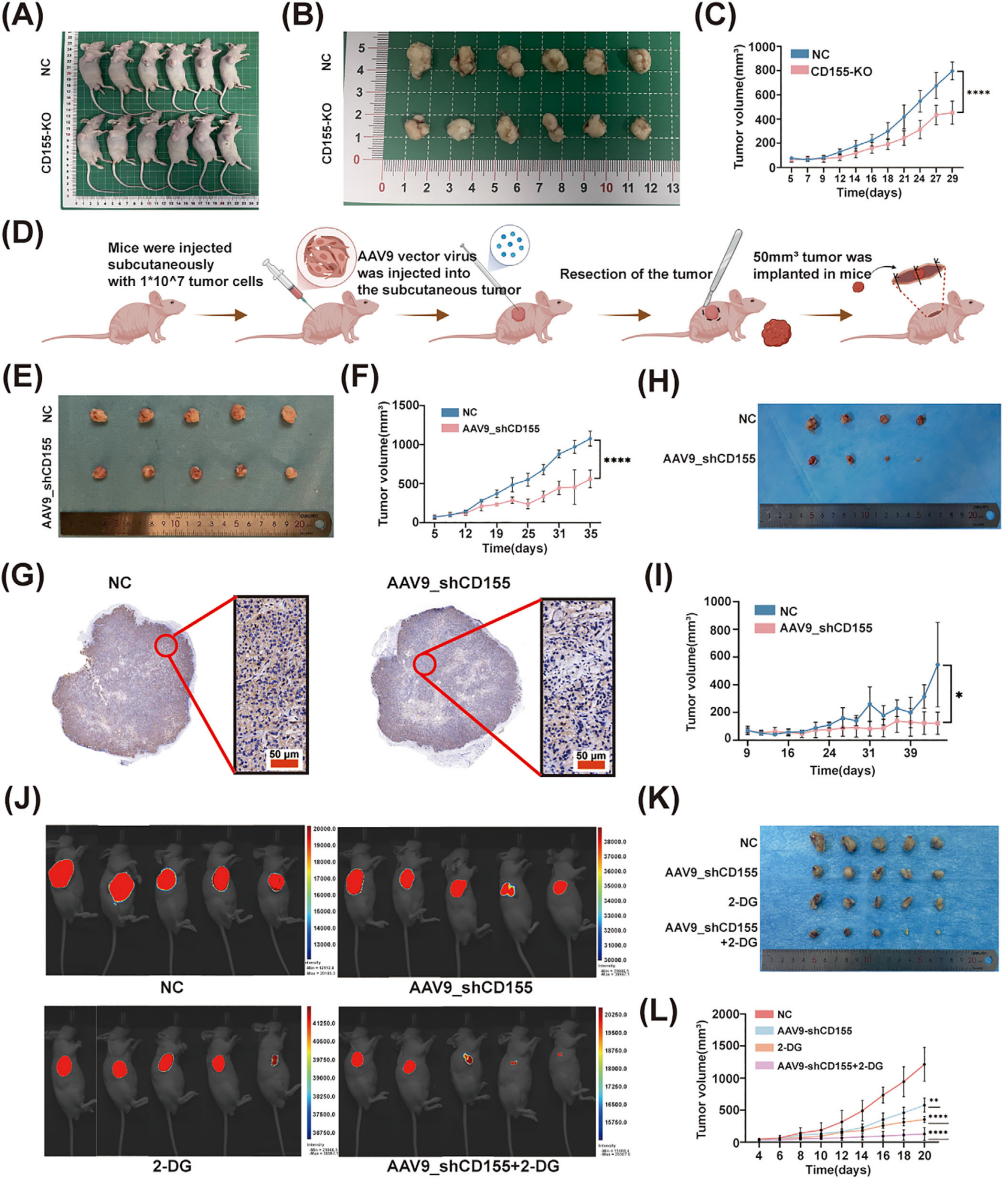
CD155 promotes the growth of NSCLC in vivo. (A–C) Subcutaneous xenograft tumours established with A549 cells were used to compare tumour growth between control (NC) and CD155‐KO groups. (D) Schematic illustrating the in vivo delivery of AAV9_shCD155. (E, F) Representative tumour images and growth curves of nude mice following AAV9_shCD155 treatment. (G) IHC staining comparing CD155 levels in tumours from the NC and shCD155 groups. (H, I) Tumour images and growth curves depicting postoperative recurrence in nude mice. (J–L) Therapeutic assessment of 2‐DG combined with AAV9_shCD155, including tumour growth monitoring. In C, F, I, and L, data are shown as mean ± SEM, and statistical significance was determined using an unpaired *t*‐test. *p < .05, ** *p* < .01, *** *p* < .001, **** *p* < .0001.

To evaluate the oncogenic function of CD155 in vivo, subcutaneous xenograft models were established using A549 cells. Tumour growth was significantly attenuated in mice implanted with CD155‐KO cells compared to those receiving control (NC) cells (Figure 6A–C).

We next evaluated the therapeutic potential of targeting CD155 in vivo. Subcutaneous tumours were established in BALB/c nude mice using A549 cells (Figure 6D). When tumours reached 100–200 mm^3^, mice received intratumoural injections of AAV9_shCD155 to achieve endogenous CD155 knockdown. Tumour growth in the AAV9_shCD155 group was significantly slower than in the NC group over 35 days (Figure 6E and F), and IHC confirmed reduced CD155 expression in the treated tumours (Figure 6G). Furthermore, in a postoperative recurrence model, local CD155 knockdown via AAV9_shCD155 also markedly impaired tumour regrowth capacity compared to the NC group (Figure 6H and I). To investigate a potential synergistic strategy, we established a syngeneic subcutaneous model using LLC cells. Mice were treated with AAV9_shCD155, the glycolysis inhibitor 2‐DG, or a combination of both. Both AAV9_shCD155 and 2‐DG alone significantly suppressed tumour growth compared to the NC group. Strikingly, the combination therapy resulted in superior tumour suppression (Figure 6J–L).

### 3.5 Anti‐CD155 effectively addresses resistance to anti‐PD‐1 therapy

**FIGURE 7:**
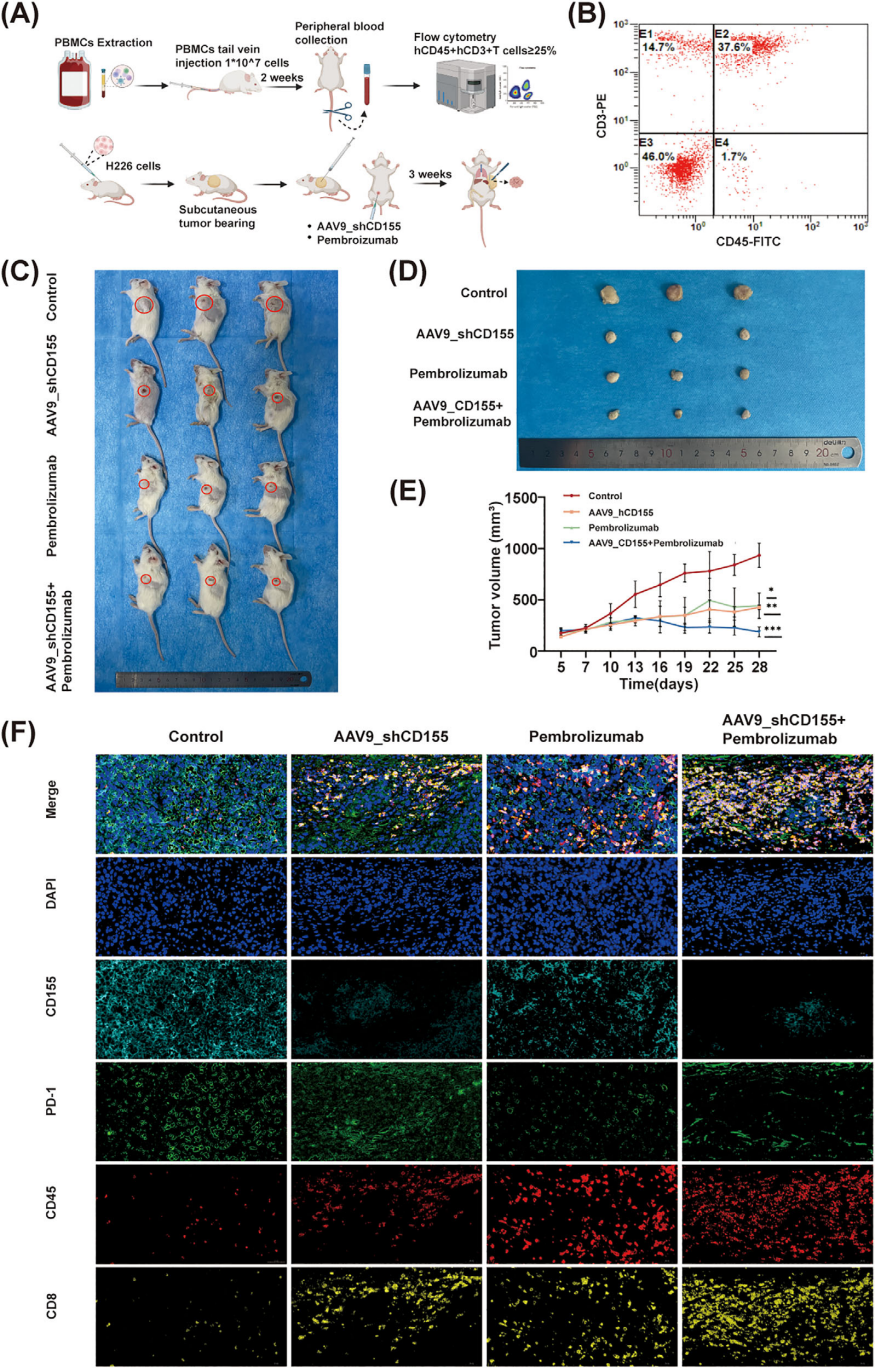
Targeting CD155 synergises with anti‐PD‐1 to inhibit tumour growth and promote immune infiltration. (A) Establishment of a humanised mouse model for subcutaneous xenograft tumour formation. (B) The content of CD3+CD45+T in peripheral blood of mice was detected by flow cytometry, CD3+CD45+T > 25%. (C, D) Mouse tumour diagram and (E) tumour growth curve. (F) Immunofluorescence staining of CD155, PD‐1, CD45, and CD8 was performed to evaluate tumour immune infiltration across different groups. In E, data are presented as mean ± SEM and statistical significance was analysed using the an unpaired *t*‐test. *p < .05, **p < .01, ***p < .001.

We next evaluated the in vivo therapeutic potential of combining CD155 inhibition with anti‐PD‑1 blockade. A humanised mouse model was established by intravenously injecting human peripheral blood mononuclear cells (PBMCs) into NCG mice (Figure 7A). Successful engraftment, defined as > 25% human CD3^+^CD45^+^ T cells in peripheral blood at two weeks, was confirmed by flow cytometry (Figure 7B). Subsequently, H226 cells were injected subcutaneously into these mice to establish tumours. Tumour‐bearing mice were randomised into four groups: Control, AAV9_shCD155 (intratumoural injection), pembrolizumab (anti‑PD‑1, intraperitoneal injection), or the combination of AAV9_shCD155 and pembrolizumab. After 28 days of treatment, all three treatment groups showed significantly slower tumour growth than the Control group. Notably, the combination therapy (AAV9_shCD155 + pembrolizumab) exhibited the most potent tumour suppression (Figure 7C–E). To further evaluate the impact of CD155 targeting on the tumour immune microenvironment, we performed multiplex immunofluorescence staining for CD155, PD‐1, CD45, and CD8 on tumour sections from the humanised mouse model (Figure 7F). Compared to the control group, both AAV9_shCD155 and pembrolizumab monotherapies significantly reduced CD155 expression and increased the infiltration of CD45^+^ leukocytes and CD8^+^ T cells. Strikingly, the combination of AAV9_shCD155 and pembrolizumab led to the greatest enhancement of CD8^+^ T‐cell infiltration. These results indicate that CD155 knockdown, particularly in combined with PD‐1 blockade, remodels the tumour microenvironment by promoting CD8^+^ T‐cell recruitment and reducing CD155‑mediated immune evasion.

## 4 DISCUSSION

Resistance to anti‐PD‐1 therapy poses a major obstacle in cancer treatment. Prior studies in glioblastoma have demonstrated that targeting the CD155 receptor TIGIT combined with anti‑PD‑1, guided by CD155 expression levels, effectively boosts T‐cell and myeloid cell activity, improving overall survival.^28^ Additionally, a retrospective study investigating the relationship between patient CD155 expression and response to PD‐1 inhibitors demonstrated that CD155 expression attenuates the effectiveness of anti‐PD‐1 treatment and increases the risk of disease progression.^29^ This finding aligns closely with our results, as patients with NMPR after anti‐PD‐1 treatment exhibited elevated CD155 expression, suggesting a link between CD155 expression and resistance to anti‐PD‐1 therapy.

This study demonstrates that high CD155 expression in NSCLC is associated with an unfavourable prognosis. Furthermore, the transcription factor ETS1 was revealed to be the upstream regulator driving CD155 overexpression in NSCLC. Multi‐omics analysis revealed that CD155 is critically involved in the malignant progression of NSCLC, mediating tumour glycolysis by upregulating key glycolytic proteins (GLUT1, GLUT3, and LDHB) through the PI3K/AKT/HIF‐1α pathway. In both in vitro and in vivo experiments, knockout of CD155 effectively impaired glucose metabolism, proliferation, and migration capabilities of NSCLC cells. These effects were particularly pronounced compared to conditions where glycolysis was inhibited, highlighting the central role of CD155‐driven glycolysis in these malignant phenotypes. In animal models, combination therapy using AAV9_shCD155 with the anti‐PD‐1 drug pembrolizumab overcame the limitations of monotherapy, effectively inhibiting tumour growth and promoting immune cell infiltration. These findings establish a strong preclinical foundation for a dual‐targeting strategy against CD155 and PD‐1 to overcome therapeutic resistance.

CD155 is a key immunomodulatory molecule in tumours. TIGIT, an immunoglobulin superfamily receptor for CD155, has attracted considerable research interest. Primarily expressed on T cells and NK cells, TIGIT binds CD155 with high affinity, and together they collectively mediate negative immune regulation in the tumour microenvironment.^30^ Freed‐Pastor et al. reported that TIGIT^+^ CD8^+^ T cells become exhausted upon encountering tumour cells with elevated CD155 expression. However, simultaneous blockade of TIGIT, PD‐1, and CD40a can reinvigorate these exhausted cells, thereby reconstituting immune function.^19^ Research in cervical cancer revealed that the binding of CD155 to phosphorylated TIGIT recruits the phosphatase SHIP‐1, which subsequently suppresses NF‐κB and ERK activation, leading to reduced cytokine production.^20^ Furthermore, He et al. demonstrated that the CD155‐TIGIT axis in gastric cancer disrupts glucose metabolism in T cells, suppressing cytokine production and facilitating tumour immune escape.^31^ Consistent with these findings, our results also show that decreased CD155 expression reshapes the immune microenvironment and enhances CD8^+^ T‐cell infiltration.

CD155 critically influences tumour metastasis, migration, and proliferation. Prior research has delineated its role in activating diverse oncogenic signalling pathways. In NIH3T3 cells, CD155 potentiates the serum‐driven Ras‐Raf‐MEK‐ERK signalling cascade, leading to the upregulation of cyclin D2 and cyclin E and the downregulation of the cell cycle inhibitor p27^Kip1^, thereby promoting tumour cell proliferation.^22^ Furthermore, in esophageal cancer, CD155 has been shown to regulate tumour proliferation and migration through modulation of the Hippo‐YAP pathway.^32^ Similarly, another study in esophageal cancer demonstrated that downregulation of CD155 suppresses both the PI3K/AKT and MAPK signalling pathways, impairing tumour growth through cell cycle arrest and apoptosis induction.^33^ In cervical cancer, CD155 associates directly with AKT, forming a complex that triggers the AKT/mTOR/NF‐κB cascade and consequently dampens both autophagy and apoptosis. Notably, excessive activation of AKT has been confirmed to be closely associated with early recurrence in various solid tumours, including lung cancer.^34^, ^35^ In this context, silencing CD155 alters the expression of cell cycle proteins and impedes tumour cell progression. Consistent with these established mechanisms across cancer types, our findings not only confirm that CD155 specifically promotes the proliferation and metastatic potential of NSCLC cells through the PI3K/AKT/HIF‐1α signalling axis, but also suggest that aberrant activation of this pathway may represent a key mechanism driving early tumour recurrence.

Recently, studies have demonstrated that immune checkpoints can alter the metabolic reprogramming of tumour cells, thereby enhancing their survival capability under nutrient‐deficient conditions within the tumour microenvironment and creating a favourable environment for tumour cell survival and malignant progression.^36^ In T‐cell lymphoma, T cells lacking PD‐1 expression induce rate‐limiting factors for glucose uptake and metabolism via the PI3K‐AKT‐mTOR‐HIF1α axis, which in turn enhances energy production in tumour cells, exacerbates T‐cell malignant transformation, and promotes tumour immune evasion.^37^ Similarly, the expression of PD‐L1 on tumour cells promotes glycolysis and activates AKT/mTOR signalling within the tumour cells themselves.^38^ In hepatocellular carcinoma, tumour cell PD‐L1 complexes with EGFR and ITGB4, activating the PI3K/AKT/mTOR/SREBP1c pathway and upregulating lipogenic enzymes (e.g., FASN, ACLY) to promote lipid metabolism and tumour growth.^39^ Correspondingly, in gastric cancer, colorectal cancer, and triple‐negative breast cancer, engagement of the TIGIT/CD155 pathway influences phosphorylation via the PI3K/AKT/mTOR cascade, leading to a significant downregulation of key glycolytic enzymes in T cells, including Glut1, HK1, HK2, and phosphofructokinase (PFK). This leads to diminished glucose uptake and lactate production, thereby compromising T‐cell activation and effector functions.^31^, ^40^, ^41^ In the present study, based on metabolomic sequencing and mass spectrometry results, we analysed the potential energy metabolism pathways affected by aberrant CD155 expression. CD155 is associated with the glycolytic pathway in tumours, a finding consistent with the analysis of TCGA data. The PI3K/AKT/HIF‐1α pathway, a central signalling route for glycolysis in tumour cells, further upregulates critical glycolytic enzymes, including GLUT1, GLUT3, and LDHB. Moreover, our data reveal a direct physical association between CD155 and the glycolytic enzyme ALDOA, suggesting a potential for more precise regulation of glycolytic flux. In summary, we have established that CD155 drives NSCLC tumour proliferation by orchestrating glycolysis. This discovery elucidates a previously unrecognised pathway that is independent of immune cells.

This study has certain limitations. First, although we established humanised mouse models and utilised xenograft models to investigate the efficacy of the combination therapy, we did not assess key parameters such as overall survival, toxicity, or adverse effects – critical factors for clinical translation. Second, the use of CD155‑knockout tumour cell lines in xenograft models helped clarify cell‑intrinsic functions but did not allow evaluation of the specific contribution of endogenous CD155 in immune cells. Future studies employing conditional knockout mouse models will be necessary to delineate the cell‑type‑specific roles of CD155 in tumour progression and therapeutic response. Third, the precise mechanisms by which CD155 mediates immune evasion in this context require further validation. Fourth, our single‑cell RNA‑seq analysis revealed an inverse expression pattern between CD155 and PD‑L1 in NMPR patients, suggesting a potential compensatory relationship. However, this observation remains descriptive, and the hypothesised feedback regulation has not been experimentally verified. Given that the primary focus of this study was to characterise CD155's role in glycolysis and anti‑PD‑1 resistance, we did not further investigate the mechanistic crosstalk between CD155 and PD‑L1. Future studies should explore whether and how PD‑L1 expression modulates CD155 transcription or stability, and vice versa, to fully understand their interplay in therapy resistance. Finally, while our combination strategy (AAV9_shCD155 plus anti‑PD‑1) showed enhanced antitumour efficacy, it was validated in different animal models and with different tumour cell lines (H226 in humanised mice for anti‑PD‑1 combination, and LLC in nude mice for 2‑DG combination) rather than in a single uniform model. Therefore, head‑to‑head comparisons under identical experimental conditions are needed to definitively establish the relative efficacy of these regimens. These limitations must be resolved through further research before clinical translation can be contemplated.

## 5 CONCLUSION

This study identifies CD155 as a critical immune checkpoint molecule driving resistance to PD‐1 blockade in NSCLC. CD155 is overexpressed in NSCLC and promotes tumour glycolysis and malignant phenotypes. Mechanistically, CD155 orchestrates these effects via the PI3K/AKT/HIF‐1α pathway, thereby driving tumour progression and conferring therapeutic resistance. Collectively, these data validate CD155 as an attractive therapeutic target, and the demonstrated preclinical activity of co‐targeting CD155 and PD‐1 inhibition provides a strong rationale for this novel combinatorial strategy.

## AUTHOR CONTRIBUTIONS

Jin‐bo Zhao, Nan Ma, and Xiao‐Long Yan conceived and supervised the project. Jin‐Bo Zhao, Xi‐Yang Tang, and Wei‐Guang Du designed the research. Wei‐Guang Du, Xi‐Yang Tang, and Yu‐Long Zhou conducted the majority of the experimental work. Zhi‐Bo Feng, Meng‐Chao Li, Jun‐Yang Pan, Yao Lv, and Xiao‐Liang Xu participated in some of the animal experiments and data analysis. Run‐Ze Zhang assisted with the analysis of public data. Wei‐Guang Du completed the writing of the manuscript and the organisation of the data.

## CONFLICT OF INTEREST STATEMENT

The authors declare that they have no competing interests.

## ETHICS STATEMENT

All patients signed informed consent forms, agreeing to the collection and use of samples as well as the use of other clinical data. All experiments performed using these samples were approved by the Ethics Committee of the Air Force Medical University.

## CONSENT

All authors consent to the content of the manuscript and to being listed as co‐authors.

## Supporting information



SUPPORTING INFORMATION

SUPPORTING INFORMATION

## Data Availability

The raw data have been deposited in Figshare and will be publicly available upon publication. The DOI is as follows: DOI:10.6084/m9.figshare.29306825. The sequencing data supporting the results of this study have been deposited in the GEO database with the accession number GSE293360.
