# Supplementary material for: Endogenous CD155 drives metabolic reprogramming via PI3K/AKT/HIF‐1α‐glycolysis axis to mediate anti‐PD‐1 resistance in non‐small cell lung cancer
Source: Clin Transl Med. 2026 Aug 10;16(8):e70741. doi: 10.1002/ctm2.70741 (PMC13458036; doi:10.1002/ctm2.70741)
Supplement: Supplementary file 1 [file CTM2-16-e70741-s002.docx]

**Supplementary Figure**

 **FIGURE S1**

(A)Approximately 92,330 cells from 15 samples were annotated based on specific markers and displayed using a bubble plot. (B and C) The CNV scores and t-SNE plots of the 17 clusters of epithelial cells. (D) Bubble plot showing the marker annotation of epithelial cells.

**FIGURE S2** Cell clustering in the six NSCLC single-cell sequencing samples.

(A) Bubble plot of subpopulation annotations for each cell. (B and C) Clustering and marker gene visualization of T cells. (D and E) Clustering and marker gene visualization of B cells. (F and G) Clustering and marker gene visualization of fibroblasts. (H and I) Clustering and marker gene visualization of endothelial cells. (J and K) Clustering and marker gene visualization of myeloid cells. (L) Bubble plot of marker annotations for epithelial cells.
